# Moving in unison after perceptual interruption

**DOI:** 10.1038/s41598-020-74914-z

**Published:** 2020-10-22

**Authors:** Benoît G. Bardy, Carmela Calabrese, Pietro De Lellis, Stella Bourgeaud, Clémentine Colomer, Simon Pla, Mario di Bernardo

**Affiliations:** 1 EuroMov Digital Health in Motion, Univ. Montpellier, IMT Mines Ales, 34090 Montpellier, France; 2 Department of Electrical Engineering and Information Technology, University of Naples Federico II, 80125 Naples, Italy

**Keywords:** Human behaviour, Perception

## Abstract

Humans interact in groups through various perception and action channels. The continuity of interaction despite a transient loss of perceptual contact often exists and contributes to goal achievement. Here, we study the dynamics of this continuity, in two experiments involving groups of participants ($N=7$) synchronizing their movements in space and in time. We show that behavioural unison can be maintained after perceptual contact has been lost, for about 7s. Agent similarity and spatial configuration in the group modulated synchronization performance, differently so when perceptual interaction was present or when it was memorized. Modelling these data through a network of oscillators enabled us to clarify the double origin of this memory effect, of individual and social nature. These results shed new light into why humans continue to move in unison after perceptual interruption, and are consequential for a wide variety of applications at work, in art and in sport.

## Introduction

Humans and other animals often cooperate in small or large ensembles, for anti-predation, for producing a collective performance, or sometimes just for entertainment. Among all sorts of cooperative behaviours, synchronization in space and/or in time of the members of the group is particularly present in the human repertoire. It is often rooted in perceptuo-motor synergies in which proximal (e.g., postures, breaths) or distal (e.g., gazes, voices, hands, legs) parts of the body are delicately locked, for brief or long periods of time, in frequency and in phase^1,2^. This is the case where a collective performance is produced, for instance in sport (e.g., team rowing or synchronized swimming^3^), in dance or in music (performing a ballet, playing in a quartet^4^), during marches and parades^5^, or even at work (collective hoeing, see^6^). In these and other examples, moving in unison is either the goal or clearly contributes to it, and results from both (i) personalized characteristics and (ii) the way individuals are coupled together.

Personalized characteristics refer for instance to mechanical properties such as body inertia, length of limbs, or location of the centre of mass. They also involve several psychological variables such as personality or pro-social attitudes, which are known to shape the way each of us spontaneously moves, as an individual or in synergy with another individual^7^. A crucial aspect of these personalized characteristics is the degree of similarity between the individuals involved, which facilitates synchronization. The identical mechanical properties of moving limbs, such as their natural oscillation frequencies, increase the level of synchronization by virtue of physical principles^8^. The morphological and kinematic resemblance of cooperating humans is also beneficial for synchronization^9,10^, which has positive effects in return for increasing emotional empathy^11^, likeability^12^, social connectedness and rapport in general^13^. This circular causality between morphological or kinematic similarity and our social behaviours can also be traced in other mammals^14^, fish and birds^15^, and even in extremely simple multi-cellular animals engaged in synchronized food foraging^16^.

**Figure 1:**
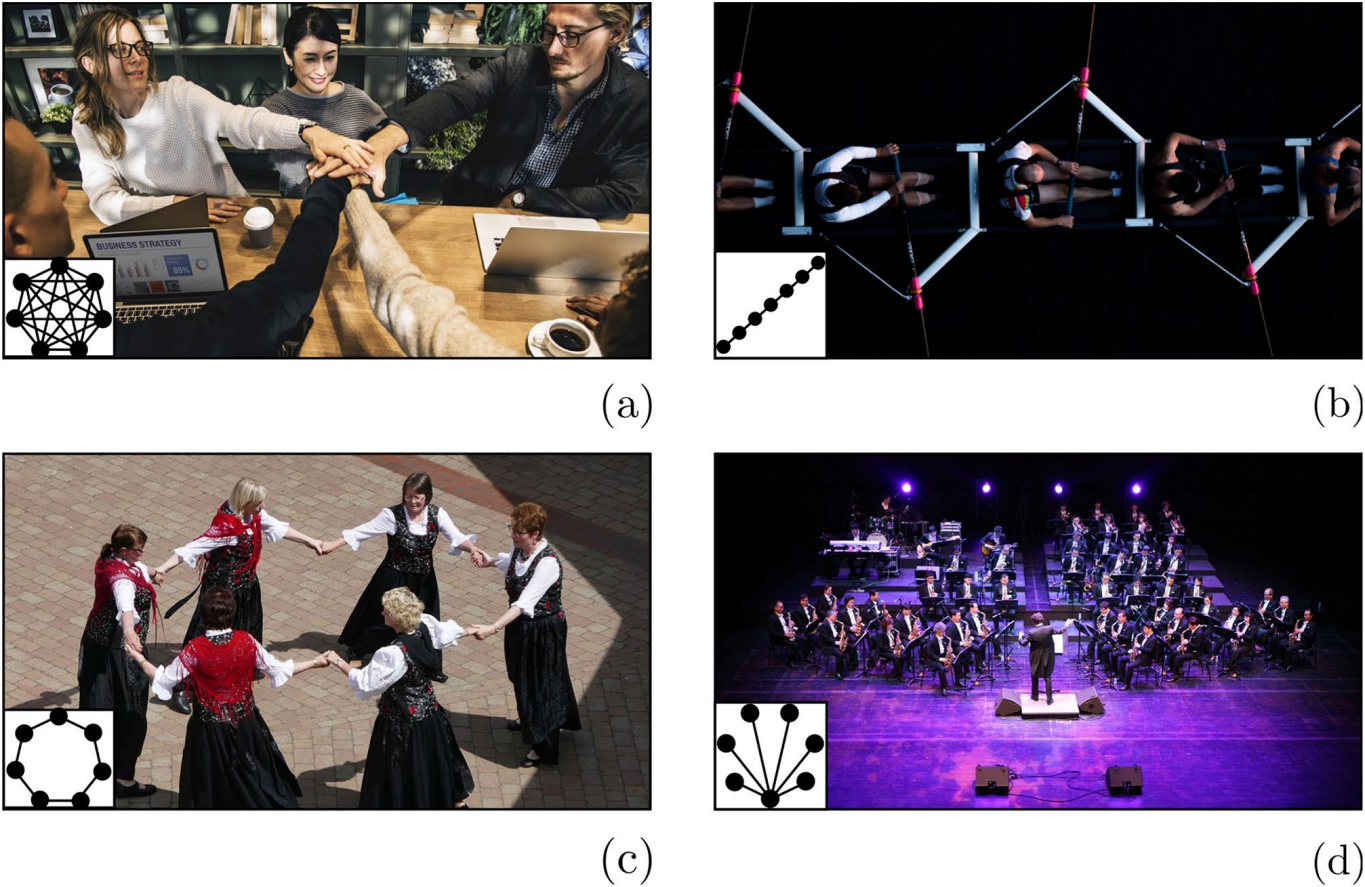
Four topologies during familiar human group cooperation situations, with various coupling modalities. (**a**) *Complete graph*: an ordinary organization during everyday working meetings; (**b**) *Path graph*: often present in sports, for instance in team rowing where partners are mechanically and visually coupled to two neighbors, except for the first and last rowers; (**c**) *Ring graph*: a common structure in many popular dances or among children at play (*round dance*); (**d**) *Star graph*: typical of musical ensembles, for instance when orchestra members are visually coupled only to the director. The image in panel (**b**) comes from unsplash.com, all the others from pixabay.com.

Synchronization also requires a coupling function between the various systems involved, whether these systems are of physical, biological, or social origin (see^17^ for a recent review). Perceptual contact is the most natural form of coupling between agents in a group. It obviously plays a crucial role, as the emergence and stability of a particular group structure heavily depend on how individuals are perceptually coupled, through mechanical^18^, optical^19^, or acoustical exchanges^20^. Mechanical coupling gives rise to fast inertial or vibratory exchanges between bodies and segments that our sophisticated proprioceptive machinery is able to detect^21^, facilitating synchronization, for instance in sport and dance (e.g., Fig. 1b and c). Visual and auditory couplings are the most pervasive forms of perceptual interaction in human groups, either separately^22^ or combined, for instance during a meeting or when playing in an orchestra^23,24^ (see Fig. 1a and d). Of interest for the present research is the recent discovery that certain topologies of the spatial organization of members in the group affect the strength and symmetry of perceptual coupling. For instance, the Complete graph (e.g., Fig. 1a) maximizes coupling strength in a symmetric manner (of the type many-to-many or N:N), while the Star graph maximizes asymmetric coupling (of the type N:1) and the emergence of leadership (e.g., Fig. 1d). Path (e.g., Fig. 1b) and Ring (e.g., Fig. 1c) graphs are also respectively symmetric and asymmetric structures, of lower coupling strength, and have been found to exhibit less cohesive behaviour in human participants engaged in visually-based synchronization tasks^25^.

In these and other examples, perceptual connection among participants is often temporarily lost. The current study targets this powerful capacity of humans to maintain regimes of synchronization despite a transient loss of perceptual (i.e., visual) coupling. This phenomenon occurs for instance when a group of people continue to walk at the same pace even after they separate, or when dancers in a choreographic performance maintain body synchronization during a transient lack of visual connection. This capacity is a solid contributor to a wide range of social performances, in sport or at work. It results from physical and neural principles, but can also be shaped by social norms^26,27^. It relies on our practical ability to internalise previously-produced movement patterns in a social context, and to maintain them when alone for a certain amount of time. The etiology of this ability is somewhat dual. One approach, the *individual memory approach*, considers this persistence effect as a witness of our capacity to prolong a movement pattern previously produced under a certain goal (intentional group synchronization) into a new context (solo action). A very large body of data from the “move-on-the beat” literature, particularly obtained in the synchronization-continuation paradigm (SCP), supports this approach, both in a solo context^28,29^ and in a dyadic context^30^. In SCP situations, participants have to move (usually tapping) to an isochronous induction beat and then maintain the beat in coordination with the (remembered) rhythm after the induction beat has stopped. Various modalities (e.g., visual or auditory) or movement types (e.g., finger tapping or whole-body movements) have been tested, and the neural circuitry dedicated to the mental simulation of the previously-induced tempo has been identified^31^. In contrast, the *social memory approach*^32,33^ suggests that persistence after visual interruption is the consequence of the mental simulation of the social interaction previously created. It also predicts that a certain proximity in individual movement frequencies fastens the route toward synchronization and helps to maintain a stronger coordinative regime during perceptual coupling and temporarily after its interruption^33^, but anchors these evidences into the social benefits of synchronization. As synchronized behaviours tend to increase social connectedness^34,35^, social feelings^36^, affiliation^27^, and cooperative behaviours in general^37^, these social benefits transiently persist once visual contact has been switched off. Neurophysiological findings show that specific oscillatory networks in the parietal cortex accompany interpersonal coordination^38^, and that human brain-to-brain synchronization tend to predict mutual prosociality^39^ and social bond^40^. These findings are compatible with the social origin of this memory effect. Besides these alternative and not necessarily exclusive explanations, the memory effect has only been experimentally studied in dyadic situations, and its dynamics in various spatial configurations remains unknown. Addressing this issue is of pressing interest, not only for basic science, but also for its potential consequences toward the acquisition and mastering of cooperative patterns in a variety of domains such as daily work, sport, or music performance.

Here we investigated the dynamics of voluntary synchronization, in groups composed of seven participants, manipulating their similarity, spatial organization, and the presence or duration of visual coupling. Participants were engaged in an intentional group synchronization task and had to swing a pendulum in order to achieve unison in space and in time (phase synchronization). This task was selected as (i) it is extremely easy to learn and perform, (ii) it has been documented before in a dyadic context^19^, and (iii) it allows a simple yet precise control over each participant’s natural frequency. Each trial started with an eyes-closed period, denoted $EC_{1}$, in which each player oscillated their own pendulum at their preferred pace. This was followed by an eyes-open period, *EO*, where they had to reach synchronization as fast as possible. The last period, $EC_2$, was again an eyes-closed sequence, split into two time intervals of equal length, denoted by $EC_{2a}$ and $EC_{2b}$, in order to better identify the possible presence and duration of a memory effect. For further details, see Supplementary Information.

Spatial organization was manipulated by rearranging participants into four group configurations corresponding to four graphs (Complete, Ring, Path, Star, see Fig. 1). In Experiment 1, participants’ similarity (i.e., homogeneity) was controlled by manipulating the pendula’s inertia and hence the natural frequency of the players’ oscillatory motion. This enabled us to evaluate the influence of the players’ similarity and graph structure on the emergence and quality of group synchronization. Specifically, four conditions were considered, involving (i) individual oscillations (*solo*), and three collective oscillations (ii) at the same shared frequency (*matched*), (iii) at the same frequency for six out of the seven players (*matched-but-one*), and (iv) at seven different frequencies corresponding to each player’s preferred pace (*natural*).

In Experiment 2, homogeneity among the players was manipulated at a different scale, by comparing groups of novices with groups of certified dancers. Ballet and ballroom dancers encounter various neural^41^, cognitive^42^ and motor^43^ changes during their years of practice, and can be considered as experts in sensorimotor synchronization compared to non-dancers^44–46^.

For homogeneity, we predicted that similarity would strengthen synchronization, irrespective of graph topology (Experiment 1), and that dancers would maintain a more solid synchronization regime compared to non-dancers (Experiment 2). For topology, we expected that Complete and Star graphs, that were observed to maximize synchronization metrics during visual contact^25^, would still be associated to higher levels of coordination after visual interruption. Furthermore, we predicted that a stronger memory effect would be present in the case of higher homogeneity between participants (similar pendulum frequencies in Experiment 1 and dancers in Experiment 2) and in graphs producing higher perceptual exchanges (Complete and Star graphs).

In addition, in order to evaluate the contribution of memory (and of which type, individual or social) to synchronization persistence after visual interruption, we developed three versions of a dynamical model capturing the essence of our experimental data, with the potential for generalization to various group situations during which perceptual contact is transiently lost. The On-Off version of the model (the Static Coupling model—**SC**) is based on a heterogeneous network of coupled Kuramoto oscillators (see Eq. 1), whose dynamics after visual interruption was evaluated. Two additional versions of the model were contrasted to better understand the origin of the memory effect following visual interruption: the individual memory (**IM**) version, and the social memory (**SM**) version. Similarly to the **SC** model, these two versions predict a decay rate in synchronization metrics when vision is removed. However, the **IM** version predicts that the decay is based on the individual motion features of each participant, whereas the **SM** version predicts a decay dependent upon the synchronization strength at the time of visual interruption (see next section for all versions of the model).

**Figure 2:**
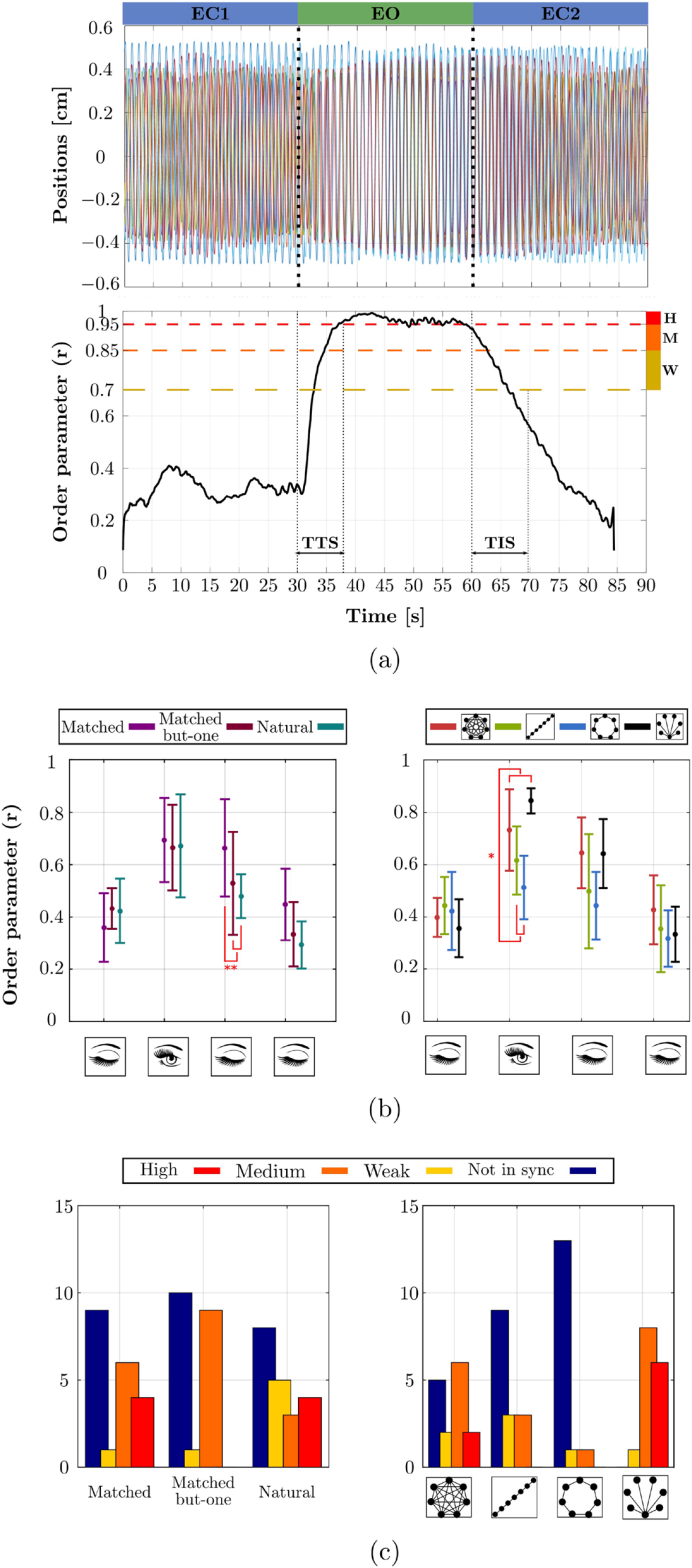
Main results of Experiment 1. (**a**) a representative example of phase synchronization *r* across periods of absence and presence of visual coupling (Time To Sync $\mathrm {TTS} = 7.49\, \text{s}$ and Time In Sync $\mathrm {TIS} = 9.78\,\text{s}$); (**b**) mean and standard deviation of phase synchronization across homogeneity (left panel) and topology conditions (right panel), $n=240$; (**c**) distribution of phase synchronization levels (High, Medium, Weak, Not in sync) for Similarity (left panel) and Topology (right panel), $n=60$.

**Figure 3:**
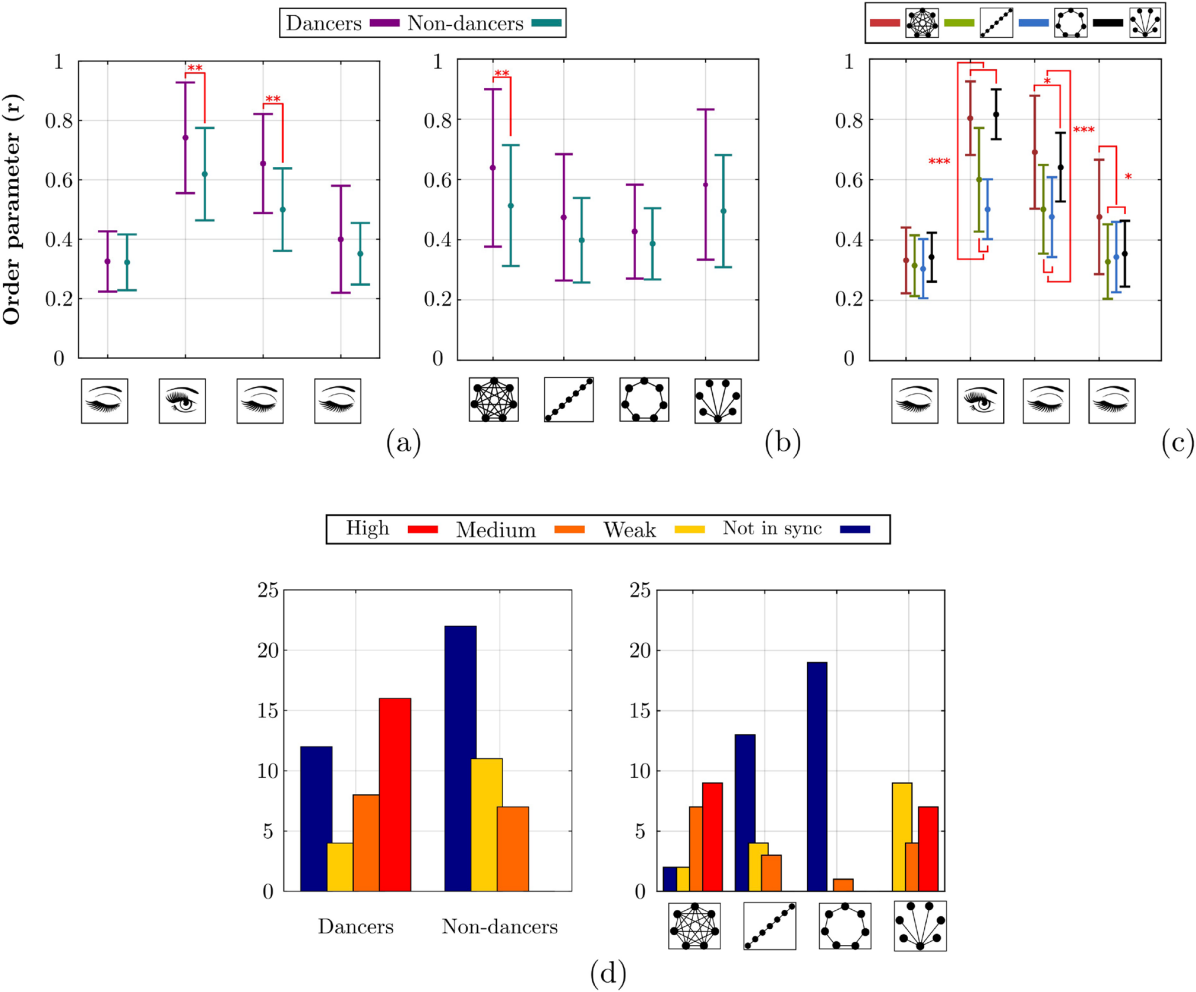
Main results of Experiment 2. Mean and standard deviation of Phase synchronization *r* in Experiment 2 as a function of (**a**) Vision $\times$ Expertise, (**b**) Expertise $\times$ Topology, (**c**) Vision $\times$ Topology, $n=320$; (**d**) distribution of phase synchronization levels across categories of robustness for Expertise (left panel) and for Topologies (right panel), $n=80$.

**Table 1:** Comparison of the Static Coupling, Individual Memory and Social Memory models with the experimental results: average (with standard deviation) experimental Time-In-Sync $\overline{\mathrm {TIS}}_{\mathrm {exp}}$ versus average (with standard deviation) simulated Time-In-Sync $\overline{\mathrm {TIS}}_{\mathrm {sim}}$; **$p<0.01$, ***$p<0.001$. See Supplementary Information for statistical details.

	Conditions	Experimental results	Static Coupling	Individual Memory	Social Memory
		$\overline{\mathrm {TIS}}_{\mathrm {exp}}$	$\overline{\mathrm {TIS}}_{\mathrm {sim}}$	$\overline{\mathrm {TIS}}_{sim}$	$\overline{\mathrm {TIS}}_{\mathrm {sim}}$
**Experiment 1**	Matched	$9.95 \pm 3.71\,\text{s}$ (n=15)	$6.52 \pm 2.88\,\text{s}$ (n=65) **	$9.73 \pm 3.72\,\text{s}$ (n=139)	$9.71 \pm 3.67\,\text{s}$ (n=123)
	Matched-but-one	$8.20 \pm 1.94\,\text{s}$ (n=10)	$5.94\pm 2.77\,\text{s}$ (n=39) **	$8.26 \pm 2.55\,\text{s}$ (n=106)	$8.16 \pm 3.23\,\text{s}$ (n=112)
	Natural	$5.32 \pm 1.17\,\text{s}$ (n=11)	$4.74 \pm 1.06\,\text{s}$ (n=12)	–	–
**Experiment 2**	Dancers	$8.81 \pm 3.42\,\text{s}$ (n=32)	$5.92\pm 2.11\,\text{s}$ (n=129) ***	$8.90 \pm 2.97\,\text{s}$ (n=251)	$8.97 \pm 3.36\,\text{s}$ (n=242)
	Non dancers	$6.26 \pm 2.43\,\text{s}$ (n=17)	$5.66\pm 2.07\,\text{s}$ (n=55)	–	–

## Results

### Experiment 1

#### Individual and group frequencies

On average, individual frequencies $\omega _{i}$ were measured to be $5.34\,\mathrm{rad}/\mathrm{s}$ (SD: 0.05), $5.23\,\mathrm{rad}/\mathrm{s}$ (SD: 0.03), $5.31\,\mathrm{rad}/\mathrm{s}$ (SD: 0.02), and $5.33\,\mathrm{rad}/\mathrm{s}$ (SD: 0.03) in Solo, Matched, Matched-but-one, and Natural conditions respectively, and were affected by our manipulation. A general Homogeneity effect was found ($F(3,12)=9.48$. $p=0.002$, $\eta ^2=0.70$) showing that swinging movements slowed down when performed in the groups. This slowing down was however observed only in the Matched condition (post-hoc Bonferroni difference between Solo—Matched $p=0.002$, Matched—Matched-but-one $p=0.02$, Matched—Natural $p=0.004$), at first sight a surprising result. However, as the homogeneous (Matched) condition was also the condition exhibiting the highest synchronization performance, both in frequency and in phase (see below), this suggests that our players modulated their behaviour in that condition, i.e., slowed down, in order to maximize perceptual coupling and increase performance (the group values reported here are those extracted from the eyes-open periods).

#### Movement similarity increases phase synchronization during and after visual interaction

In general, the average phase synchronization index *r* (the order parameter) ranged between 0.13 and 0.94, depending on conditions. Figure 2a illustrates the dynamics over time of *r* in a representative trial, showing how the periods of synchronization coincided with the presence of visual coupling.

The three way (Homogenity $\times$ Topology $\times$ Vision) repeated-measures ANOVA on Fisher *z*-transformed *r* values, with all degrees of freedom corrected using the Greenhouse-Geisser estimate of sphericity (see Supplementary Information), showed a main effect of Vision ($F(1.17,4.67)=53.4$, $p< 0.001$, $\eta _p^2= 0.93$), indicating that visual coupling induced phase synchronization. This main effect was completed by a Homogeneity $\times$ Vision interaction ($F(1.89,7.54)= 7.52$, $p= 0.017$, $\eta _p^2= 0.65$), see Fig. 2b (left panel), suggesting that movement similarity increased the visual advantage. Particularly interesting for the present research is the transient persistence of phase synchronization, after vision has been removed ($EC_{2a}$ condition). This is witnessed by significant post-hoc Bonferroni comparisons between $EC_1$ and $EC_{2a}$ ($p=0.001$), and between $EC_{2a}$ and $EC_{2b}$ ($p<0.001$). This was the case in all homogeneity conditions, with a clear advantage of fully similar movements compared to the other two homogeneity types ($p<0.001$). In short, group phase synchronization persisted for around 7s after visual interaction had been interrupted, a persistence that was strongly reinforced when the participants’ motion was homogeneous.

#### Topology modulates phase synchronization and its persistence after visual interruption

The ANOVA presented above also revealed an effect of Topology ($F(3,12)=9.54$, $p= 0.002$, $\eta _p^2=0.70$), showing that Complete and Star graphs yielded higher synchronization than Ring and Path graphs (post-hoc Bonferroni comparisons: Ring different from Complete, $p=0.007$, and from Star, $p=0.004$). This confirmed a result previously obtained for different types of movement^25^. More important is the finding that phase persistence after visual interruption was reinforced for the two leading topologies (Complete and Star graphs) compared to the Ring and Path graphs (see Fig. 2b, right). This is shown by the significant Topology $\times$ Vision interaction ($F(2.23, 8.93)= 10.8$, $p= 0.004$, $\eta _p^2=0.73$), and by the subsequent post-hoc analyses (Complete and Star topologies differ from Path and Ring topologies in *EO* ($p<0.001$) and in $EC_{2a}$ ($p<0.001$); Complete topology differs from Star ($p=0.02$), from Path ($p=0.01$), and from Ring topologies ($p=0.03$) in $EC_{2b}$).

#### Synchronization quality through the order parameter *r*

To assess the quality of synchronization among conditions, we defined four levels of coordination based on the order parameter values (see Supplementary Information for details): (i) not-in-sync ($r< 0.7$), (ii) weak ($0.7 \le r < 0.85$), (iii) medium ($0.85 \le r < 0.95$), and (iv) high ($0.95 \le r \le 1$). In order to test for Homogeneity and Topology effects, we ran the log-likelihood version of $\chi ^2$, the $G$-test, on the distribution of these four levels, encoded by a variable denoted by $\mathrm {Level}_r$. We found that the distributions were indeed different. Homogeneity exhibited weak synchronization when an outlier was present in the group (matched-but-one condition) ($G^2(6)=14.08$, $p= 0.02$, Cramer’s $V=0.34$) (Fig. 2c, left panel). Topology showed an increase in occurrence of medium and high phase values for the Complete and Star graphs ($G^2(9)=41.15$, $p<0.001$, Cramer’s $V=0.48$) (Fig. 2c, right panel).

#### Time-To-Synchronization (TTS) and Time-In-Synchronization (TIS)

To complete our analyses, we evaluated the effect of homogeneity in individual frequencies on the temporal aspects of the various synchronization regimes. This was performed by focusing on two variables, (i) the time to synchronization (*TTS*), capturing the time necessary for all participants to reach phase synchronization once they had opened their eyes, and (ii) the time remaining in synchronization (*TIS*) after eye closure, quantifying the memory effect (see Fig. 2a and SI Appendix section for details). *TTS* did not differ between conditions but *TIS* did, showing a group effect ($F(2, 19.66)= 15.30$, $p<0.001$, $\eta _p^2=0.61$), more precisely a difference between the Natural condition ($5.32\,\text{s}$) and the two other conditions, Matched ($9.95\,\text{s}$, $p=0.001$) and Matched-but-one ($8.20\,\text{s}$, $p=0.003$).

### Experiment 2

While Experiment 1 manipulated similarity between participants at the fast temporal scale of pendulum dynamics, Experiment 2 investigated similarity at a much more extended temporal scale. The group synchronization metrics were compared between novice and expert dancers, again across topologies and visual interaction.

#### Complete and Star graphs increased synchronization and persistence

Here as well we evaluated the synchronization performance reached by our participants in the group through the order parameter reflecting phase synchronization. In general, *r* ranged between 0.13 and 0.98, exhibiting large differences between conditions and groups. As the Mauchly’s test indicated non sphericity of the (Fisher *z*-transformed) values of *r* for Topology ($\chi ^2(5) = 17.6$, $p= 0.004$) and for the Topology $\times$ Vision interaction ($\chi ^2(44) = 64.2$, $p=0.03$), degrees of freedom were corrected using the Greenhouse-Geisser estimate of sphericity (Topology: $\epsilon = 0.59$, Topology $\times$ Vision: $\epsilon = 0.54$). The ANOVA revealed a main effect of Topology ($F(1.78,32.1)=27.8$, $p<0.001$, $\eta _p^2=0.61$), again suggesting that the Complete and Star graphs increased synchronization by about $15\%$. It also revealed a general vision effect ($F(3,54)= 196$, $p<0.001$, $\eta _p^2=0.92$), suggesting a clear memory effect for both samples of participants during the first $15\,\text{s}$ following visual occlusion ($EC_{2a}$). Interestingly, the Topology $\times$ Vision interaction (Fig. 3c) was significant ($F(4.83,87)= 14.7$, $p<0.001$, $\eta _p^2= 0.45$), indicating that this memory effect was prolonged after $15\,\text{s}$ for the Complete graph, i.e., during $EC_{2b}$ (all post-hoc $p<0.03$).

#### Expertise increased synchronization and persistence

A main effect of Expertise was also found ($F(1,18)=34.2$, $p<0.001$, $\eta _p^2= 0.66$), indicating that dancers were in general more synchronized than novices, a clear anticipated effect of expertise visible in this simple pendulum oscillation task. More importantly for our research is the significant interaction found between expertise and the other factors (Fig. 3a–c). First, the Expertise $\times$ Vision interaction ($F(3,54)= 16.7$, $p<0.001$, $\eta _p^2= 0.48$), Fig. 3a, revealed that dancers exhibited a higher phase synchronization in *EO* (post-hoc $p=0.002$) and in $EC_{2a}$ than non dancers (post-hoc $p=0.002$), the two conditions revealing the memory effect. Second, the Expertise $\times$ Topology interaction ($F(1.78, 32.1)=3.63$, $p= 0.04$, $\eta _p^2=0.17$), Fig. 3b, revealed that the advantage of the complete graph at facilitating group synchronization benefited more to the dancers than to the novices (post-hoc $p=0.002$).

#### Synchronization quality analysis through the order parameter *r*

As in Experiment 1, we evaluated the extent of phase synchronization by grouping the values of the order parameter into four levels (from absent to strong synchronization), and compared the obtained distributions of $\mathrm {Level}_r$ values using $\chi ^2$ tests. The analysis confirmed the general results obtained in Experiment 1. Indeed, distributions were different for Expertise ($\chi ^2(3)=22.28$, $p<0.001$, Cramer’s $V=0.53$), with a higher occurrence of poor synchronization levels for novices compared to experts (Fig. 3d, left panel). They were also different for Topology ($G^2(9)=77.29$, $p<0.001$, Cramer’s $V=0.57$) (Fig. 3, right panel), showing higher synchronization levels for Complete and Star graphs when compared to Ring and Path graphs.

#### Time To Synchronization (*TTS*) and Time In Synchronization (*TIS*)

Here again, we observed that *TTS* did not differ between groups. However, dancers were found to remain synchronized for a longer time interval after visual occlusion (in $EC_{2a}$) compared to non-dancers, $8.81\,\text{s}$ and $6.26\,\text{s}$ respectively ($U= 143$, $p= 0.007$, $r= 0.39$), consistent with the memory effect observed above.

## Modelling group synchronization and memory effect

Here we provide a modelling framework to capture the experimental observations and to test different hypotheses in order to explain the persistence of synchronization observed in groups when visual coupling is suddenly lost. Further details on models and analyses can be found in Supplementary Information. In a first attempt, following previous work^25^, we modeled the group dynamics as a network of Kuramoto oscillators, coupled through the graph topologies used in the experiment. We modeled the transition between ‘eyes closed’ and ‘eyes open’ periods by setting the coupling gain *c* instantaneously to zero so that the motion of each player in the group is modeled as

1 $$\begin{aligned} \dot{\theta }_i(t)= {\left\{ \begin{array}{ll} \omega _i+c\sum \nolimits _{j=1}^{N} a_{ij}\sin {(\theta _j(t)-\theta _i(t))}, &{} \text{ if } \text{ eyes } \text{ open },\\ \omega _i, &{} \text{ if } \text{ eyes } \text{ closed }, \end{array}\right. } \end{aligned}$$

where *N* is the number of players, $\theta _i$ the phase of the movement of the *i*-th player, $\omega _i$ their natural frequency, and *c* the strength of the coupling with the other players when visual coupling was established. The coefficients $a_{ij}$ are set equal to 1 if the topology being studied involves a visual connection between players *i* and *j* when eyes are open, otherwise they are set equal to 0. In the following, we will refer to this model as the **Static Coupling** model (**SC**).

To test the model validity, we parameterized the model from experimental data as described in Supplementary Information, and then computed the average $\mathrm {TIS}$ after switching the coupling *c* to zero. We observed that the **SC** model was unable to capture the relatively longer $\mathrm {TIS}$ measured experimentally, with model predictions being consistently shorter than expected in all conditions except in the natural condition. Significant differences were indeed found between data and simulations for Matched ($U=222$, $p=0.001$, $r=-0.37$) and Matched-but-one conditions ($U=75$, $p=0.003$, $r=-0.43$), while the model agreed with the data in the Natural condition ($U=43$, $p=0.17$, $r=-0.30$). When used to explain the observations in Experiment 2, the same model did capture the synchronization dynamics of the non-dancers ($U=405$, $p=0.41$, $r=-0.01$). However, it failed to capture the longer $\mathrm {TIS}$ exhibited by the dancers’ group during the experimental trials ($U=917$, $p<0.001$, $r=-0.38$). Therefore, a more sophisticated model is required to adequately capture the experimental observations (Table 1).

More specifically, the longer $\mathrm {TIS}$ exhibited in both experimental scenarios suggests that some memory mechanism was present, allowing the groups to stay in sync for longer than predicted by a sudden memory-less transition from eyes-open to eyes-closed. As presented in the Introduction, we contrast below two possible alternatives to model [1].

In the first model extension, the **Individual Memory** model (**IM**), we assume that the motion frequency exhibited by each player at time $t_a$ of visual occlusion remains first as similar as possible to the last frequency $\dot{\theta }_i(t_a)$ exhibited with eyes open, and then, after some time lag, relaxes back to the natural frequency of the player, $\omega _i$. The model then becomes:

2 $$\begin{aligned} \dot{\theta }_i(t)= {\left\{ \begin{array}{ll} \omega _i+c\sum \nolimits _{j=1}^{N} a_{ij}\sin {(\theta _j(t)-\theta _i(t))}, &{} \text{ if } \text{ eyes } \text{ open },\\ \omega _i+\phi (t)(\dot{\theta }(t_a)-\omega _i),&{} \text{ if } \text{ eyes } \text{ closed }, \end{array}\right. } \end{aligned}$$

with $\phi (t)=\exp \left( -(t-t_a)/\tau \right)$; $\tau$ being the estimate of the decay time observed experimentally once visual contact among the participants is lost.

We contrasted the model above with the predictions of a different model, the **Social Memory** model (**SM**). In this model, we assume that participants maintain longer synchronization times at eye closure by internalising the aggregate group dynamics. These dynamics are captured by the modulus $r_i(t)$ and phase $\psi _{\mathrm {ref}}^i(t)$ of the local order parameter computed by player *i*, using information received from their visually coupled neighbours before closing their eyes. In this case we have

3 $$\begin{aligned} \dot{\theta }_i(t)= {\left\{ \begin{array}{ll} \omega _i+c\sum _{j=1}^{N} a_{ij}\sin {(\theta _j(t)-\theta _i(t))}, &{} \text{ if } \text{ eyes } \text{ open },\\ \omega _i+c\phi (t)r_i(t_{a}) \sin {(\psi _{\mathrm {ref}}^i(t)-\theta _i(t))},&{} \text{ if } \text{ eyes } \text{ closed }, \end{array}\right. } \end{aligned}$$

where $\psi _{\mathrm {ref}}^i(t)=\dot{\psi }_{\mathrm {ref}}^i(t_{a})(t-t_a)+\psi _{\mathrm {ref}}^i(t_a)$ and $\phi (t)$ is the decay function defined above.

Both the **IM** model ($M=2.05$, $SD=1.58$ error across topologies) and the **SM** model ($M=1.38$, $SD=1.84$ error across topologies) were found to capture the experimental data (mean difference$=-0.67$, $t(6)=-1.98$, $p=0.09$, $r^2=0.40$, $95\%$ CI:$[-1.49,-0.16]$). In Experiment 2, the **IM** model ($M=0.96$, $SD=1$ error across topologies) was found to better capture the experimental data than the **SM** Model ($M=1.74$, $SD=1.53$ error across topologies, mean difference$=-0.79$, $t(7)=-2.87$, $p=0.02$, $r^2=0.54$, $95\%$ CI:$[-1.43,-0.14]$). This would explain the residual synchronization found in dancers.

## Discussion

We showed that our ability to move in unison is strongly influenced by our spatial configuration, similarity in behaviour, expertise and amount of visual exchange. In two experiments in which these factors, as well as their key interactions, were manipulated, we demonstrated that Complete and Star graphs were the most solid topologies prone to facilitating synchronized behaviours, reinforced by inertial homogeneity between participants and their expertise in perceptuo-motor synchronization. Importantly, we also demonstrated that group synchronization can be maintained for a certain amount of time (about 7 s) after informational exchanges have been interrupted, again more so in the two dominant topologies, and in a stronger way for experts. We investigated the origin of this effect by modelling our behavioural results with a simple ON–OFF dynamical model consisting in switching off the visual coupling and letting the individual dynamics relax to the initial oscillation frequency. This **Static Coupling** model was sufficient to partially capture our data. However, a memory effect had to be introduced in the model to account for the marked persistence of synchronization in eyes closed for two of the three homogeneity conditions, as well as for the coordination experts. An advantage was found in this population for the **IM** version compared to the **SM** version of the model. Complementary modelling routes have been followed using the Haken–Kelso–Bunz (HKB) model, with two^33^ or eight^47^ participants, without however the current comparison between **SC**, **IM**, and **SM** versions. Taken altogether, these results help to better understand why behavioural cohesion is easier to maintain when perceptual exchanges are lost, more so in Complete and Star spatial configurations, and how perceptuo-motor expertise can reinforce this cohesion.

Obviously, natural situations such as those described in Fig. 1 are far richer than the pendulum experiments performed here, both on the action side and on the perception side. On the action side, the task used in this study was extremely simple and does not fully reflect the dynamics of natural group cooperation situations in which participants often produce different movements, along different dimensions, with different effectors, and sometimes with different sub-goals. On the perception side, natural situations do not necessarily involve a one-to-one correspondence between topology and the type of perceptual coupling. One important reason is that virtually all natural situations involve the congruent contribution of multiple senses^48^, and different topologies can co-exist when more than one type of coupling is taken into account. While the *Meeting* example (Fig. 1a) is a complete graph, simultaneously for haptic, visual, and potentially auditory exchanges, the other examples are not quite as straightforward. The *Rowing* example (Fig. 1b) corresponds to a path graph for vision and haptics, but to a complete graph for audition; the *Round* example (Fig. 1c) to a ring graph for haptics but to a complete graph for vision and audition; and the *Orchestra* example (Fig. 1d) to an even more complex graph where vision and audition interact. How these multiple and co-existing configurations, within and across our senses, modulate our collaborative behaviours, in those and other situations, remains largely unknown and constitute a promising avenue for future research. Contrasting the **SC**, **IM**, and **SM** modelling strategies in a variety of situations, where these perceptual and topological parameters are manipulated, would help to better characterize their respective contribution and their possible complementary nature.

## Methods

Both studies were carried out according to the principles expressed in the Declaration of Helsinki, and were approved by the EuroMov ethical committee (EuroMov IRB #1811A and #1801A, University of Montpellier). All participants provided their written informed consent to participate in the study, and this consent was also approved by the ethical committee. In addition, all participants gave their informed consent for publication of identifying images (i.e., Fig. S1) in an online open-access publication.

**Participants.**

- **Experiment 1.** A group of 7 participants, selected among 30 tested students at the University of Montpellier (see below), took part in the experiment (5 males, 2 females, all right-handed; mean age $21.2\,\text{y}\pm 1.5\,\text{y}$). They had no expertise in sensori-motor synchronization activities (e.g. music or dance).
- **Experiment 2.** A total of 28 right-handed volunteers were recruited among students at the University of Montpellier. They were divided into two macro-groups according to their dancing experience. The dancers (**D**) had had more than 5 hours of practice per week over the past 10 years, and all possessed the French Dance EAT certificate (Dance professorship). The non-dancers (**ND**) had performed physical activities or sport less than three hours per week, and had never practiced dance or music before. Participants were assembled in four groups of seven individuals each. Specifically,
  - Two groups of dancers: **D1** (5 females, mean age 25.6 y ± 3.1 y) and **D2** (6 females, mean age 22.4 y ± 2.4 y);
  - Two groups of non-dancers: **ND1** (3 females, mean age 20 y ± 2.6 y) and **ND2** (4 females, mean age 21.9 y ± 2.27 y).

**Task and conditions.** In both experiments, the volunteers, seated in a circle in a quiet room with no distractions, were asked to oscillate a pendulum, in synchronization with each other (Fig. S1 in Supplementary Information). The instruction was “*Synchronize the movement of your pendulum back and forth with the movement of the others, as naturally as possible, as if you could do it for 30 minutes*”. A demonstration was performed to make sure that the task was understood by each participant, and to clarify that synchronization in phase, and not only in frequency, was expected.

Each group performed the experiments in four different interaction patterns among players (i.e., *topologies*), implemented through the combination of the spatial location of each participant and the use of home-made goggles limiting the field of vision to the desired location. Namely, the four topologies were **Complete graph**, **Path graph**, **Ring graph** and **Star graph**, see Supplementary Information for further details.

In each topology, each group performed 5 trials of $75\,\text{s}$ each in Experiment 1, and of $90\,\text{s}$ each in Experiment 2. The trials alternated absence or presence of visual contact as follows:

- **Eyes-closed period 1** ($EC_1$). The participants were asked to first swing the pendulum with their preferred hand at their own comfortable tempo during $15 \, \text{s}$ (Experiments 1) or $30 \, \text{s}$ (Experiments 2), while keeping their eyes closed.
- **Eyes-open period** (*EO*). Once the baseline was established in $EC_1$, the participants were instructed to open their eyes, synchronize their pendulums, and maintain this synchronization regime during $30\,\text{s}$;
- **Eyes-closed period 2** ($EC_2$). The participants were then instructed to close their eyes again, and to keep on swinging their pendulum for $30\,\text{s}$. For the subsequent analyses, the eyes-closed period 2 is split into two periods of equal length, denoted by $EC_{2a}$ and $EC_{2b}$, respectively.

An acoustic signal notified the requested change between visual conditions. The comparison between the first two conditions allowed us to evaluate the role of perceptual contact in creating synchronization patterns. The comparison between the two eyes-closed periods (before vs. after visual exchange) allowed the evaluation of the transient persistence of synchronization in the absence of visual contact.

In Experiment 1, these analyses were repeated in three different *Homogeneity* conditions. Indeed, the 7 participants were selected among 30 volunteers based on pre-tests that were run to compute their natural frequencies. Specifically, each participant was asked to sequentially oscillate 7 pendulums characterized by 7 different eigenfrequencies ($4.71\,\mathrm{rad}/\mathrm{s}$, $4.78\, \mathrm{rad}/\mathrm{s}$, $4.84\, \mathrm{rad}/\mathrm{s}$, $4.96\, \mathrm{rad}/\mathrm{s}$, $5.03\, \mathrm{rad}/\mathrm{s}$, $5.09\, \mathrm{rad}/\mathrm{s}$, and $5.15\, \mathrm{rad}/\mathrm{s}$, respectively), obtained by adding additional masses of $96\, \text{g}$ at different locations of the pendulums’ arm (see Fig. S1b). For each pendulum, 5 trials of $20 \,\text{s}$ each were performed to select the 7 participants of Experiment 1, based on their natural movement frequency and their stability across time and trials. This enabled the design of the three *Homogeneity* conditions:

- **Matched.** By appropriately placing the additional masses, the different natural frequencies of the 7 participants were compensated so that their swinging frequency coincided ($5.34\, \mathrm{rad}/\mathrm{s}$). The selected value of $5.34\,\mathrm{rad}/\mathrm{s}$ corresponds to the group average of all the individual frequencies recorded during the pre-tests.
- **Matched-but-one.** The six most stable participants at pre-tests (characterized by an individual coefficient of variation in the range 0.33–2.75%) were set at the same swinging frequency of $5.34\,\mathrm{rad}/\mathrm{s}$, while the seventh participant (the most unstable, coefficient of variation $4.50\%$) was set at a different frequency ($6.28\, \mathrm{rad}/\mathrm{s}$). This condition was used to test to what extent and under which interaction topology the introduction of one outlier would destabilize an otherwise homogeneous network.
- **Natural.** Here, all the additional masses were removed, and the 7 participants performed the task at their own natural frequency, ranging from $5\,\mathrm{rad}/\mathrm{s}$ to $6.13\, \mathrm{rad}/\mathrm{s}$.

**Data processing.** Each pendulum was equipped with a calibrated analog potentiometer to record its angular motion at $f_s=200\, \text{Hz}$. The acquisition was performed using the Matlab software, recording the signals of the seven pendulums simultaneously. The position time series were then smoothed out through a Moving Average filter with a time window of 10 samples ($\Delta t_w= 0.05\, \text{s}$). The Hilbert transform method was applied on the filtered positions to extract the time series of the phases.

**Data analysis and relevant metrics.** Denoting *T* as the number of samples in each trial and *N* as the number of players, we can define $\theta _i(k)$ as the phase of the *i*-th pendulum at the *k*-th sampling instant, for all $i=1,\ldots ,N$ and $k=1,\ldots ,T$. The following set of metrics were used to capture the relevant features of the human group interactions recorded in our experiments:

- **Individual frequencies and group frequency.** At each time step, we computed the angular velocity of each player by applying finite differences (*forward Euler method*) to the extracted phases:
  4 $$\begin{aligned} \omega _i(k)= \frac{\theta _i(k+1)-\theta _i(k)}{\Delta t}, \qquad i=1,2,\ldots ,N \quad , \end{aligned}$$
  with $\Delta t=1/f_s$ being the sampling time. This allowed us to characterize the frequency of each participant and its stability. Then, the average frequency of the group, $\omega _{\mathrm {group}}(k)$, was extracted as the time-average of $\omega _i(k)$.
- **Group synchronization metrics** To quantify and characterize the level of synchronization among the players, we used the following metrics:
  - *Phase-synchronization*: for each trial, we evaluated the extent of synchronization in the group at each sampling time *k* through the order parameter *r*(*k*), defined as
    5 $$\begin{aligned} r(k)=\left| \frac{1}{N} \sum _{i=1}^{N} e^{j\theta _{i}(k)}\right| \qquad \forall k \in \{1,\dots ,T\}, \end{aligned}$$
    where *j* is the imaginary unit. Note that *r*(*k*) belongs to the interval [0, 1], and it is 1 when the phases coincide at time *k*. Then, we computed the average order parameter in the trial $\bar{r}$, and that is,
    6 $$\begin{aligned} \bar{r}=\frac{1}{T} \sum _{k=1}^{T} r(k). \end{aligned}$$
  - *Levels of group phase synchronization*: to allow for a proper comparison of the extent of synchronization in the group in the various conditions introduced in the main document, we discretized the order parameters into four *phase-synchronization levels* (see Fig. S2):
    7 $$\begin{aligned} \mathrm {Level}_r(k)=\left\{ \begin{aligned}&1,\text { if }r(k)<0.70 \qquad \quad \, \mathbf{~(not~in~sync) },\\&2, \text { if }0.70\le r(k)< 0.85 \mathbf{~(weak~synchronization) },\\&3, \text { if }0.85\le r(k)< 0.95 \mathbf{~(medium~synchronization) },\\&4, \text { if }0.95\le r(k)\le 1\quad \ \mathbf{~(high~synchronization) }. \end{aligned}\right. \end{aligned}$$
    Note that $\mathrm {Level}_r(k)=1$ (not in sync) means that the phase of the pendula at time *k* cannot be grouped in a circular sector of angle $\pi \, \text{rad}$.
  - *Time-To-Synchronization and Time-In-Synchronization*: Figure S2 in Supplementary Information illustrates how data were classified in order to compute the *Time-To-Synchronization* (**TTS**) and the *Time-In-Synchronization* (**TIS**).
    - **Eyes-open** *(EO)***: computing** $\mathrm {TTS}$. For a given trial, we denote $T_{\mathrm {sync},i}$ as the number of sampling instants *k* such that $\mathrm {Level}_r(k)=i$, and the corresponding fraction $F_{\mathrm {sync},i}=T_{\mathrm {sync},i}/T$, for $i=1,\ldots ,4$. We computed $\mathrm {TTS}$ only for trials in which $F_{\mathrm {sync},1}\le 0.5$ in order to exclude from the analysis the trials in which synchronization was only occasionally achieved. The remaining trials were classified as follows:
      1. If $F_{\mathrm {sync},2} + F_{\mathrm {sync},3}> 0.75(1-F_{\mathrm {sync},1})$, then the trial was considered as an instance of *Medium* synchronization;
      2. If $F_{\mathrm {Sync},3} + F_{\mathrm {Sync},4}> 0.75(1-F_{\mathrm {sync},1})$, then the trial was considered as an instance of *High* synchronization;
      3. If neither condition 1 nor 2 are satisfied, then the trial is considered as an instance of *Weak* synchronization.

Depending on the above classification, $\mathrm {TTS}$ was defined as the first time instant such that $\mathrm {Level}_r$ became 2 (for trials of weak synchronization), 3 (for trials of medium synchronization), or 4 (for trials of high synchronization).

- **Eyes-closed** ($EC_2$): **computing** $\mathrm {TIS}$. $\mathrm {TIS}$ was the first time instant such that $\mathrm {Level}_r=1$ if the players stayed in sync ($\mathrm {Level}_r>1$) after closing their eyes for at least 3 consecutive periods of length ${2\pi }/{\omega _{\mathrm {group}}}$, where $\omega _{\mathrm {group}}$ is the mean frequency of the players in the trial. Otherwise, we set $\mathrm {TIS}=0$.

**Statistical analyses.** All statistical analyses were performed using Matlab R2016a (Mathworks), SPSS 23 (IBM) and Statistica 7.1 (StatSoft). For all metrics, we used Shapiro-Wilk tests to check the normality assumption, the Levene’s test to verify variance homogeneity, and the Mauchly test for sphericity (Greenhouse-Geisser correction was applied in the cases where sphericity was not met). Statistical analysis methods were chosen according to the verified assumptions. All statistical tests performed were two-sided. In the following, we detail the statistical analyses performed for each experiment:

- ***Individual and group frequencies.*** This comparison was conducted only for Experiment 1. We computed the average $\omega _{\mathrm {group}}$ recorded during the interaction across the three Homogeneity conditions and compared them with the average individual frequency, which we called $\omega _{\mathrm {solo}}$, that the players displayed during the pre-test session (ANOVA with factor levels [Solo, Matched, Matched-but-one, Natural], Post-hoc Bonferroni tests for pairwise comparisons).
- ***Order parameter.*** Since $\bar{r}\in [0,1]$, a Fisher *z* transformation was applied to each value before performing statistical tests.
  1. The values recorded in the 5 trials performed by the group in each condition were considered independent samples in a within-subjects analysis of variance (repeated-measures ANOVA) with *Homogeneity* [Matched, Matched-but-one, Natural], *Topology* [Complete, Path, Ring, Star] and *Vision* [$EC_1$, *EO*, $EC_{2a}$, $EC_{2b}$] as factors
  2. A preliminary analysis of the *Group* factor [**D1**, **D2**, **ND1**, **ND2**] showed that the two groups of dancers (and of two non-dancers) were not statistically different and, for this reason, the sub-groups were combined to form the *Expertise* factor [$\mathbf{D }=\{\mathbf{D1 } \cup \mathbf{D2 }\}$, $\mathbf{ND }=\{\mathbf{ND1 } \cup \mathbf{ND2 }\}$]. Therefore, the values recorded in the 10 trials performed by each group (**D**/**ND**) in each *Vision* and *Topology* condition were considered as independent samples in a mixed repeated-measures analysis of variance with one between factor — *Expertise* [Dancers/Non dancers]—and two within-factors—*Topology* [Complete, Path, Ring, Star], and *Vision* [$EC_1$, *EO*, $EC_{2a}$, $EC_{2b}$].
- $\mathrm {Level}_r$. The following analyses were performed to test the effects of the factors on the synchronization levels:
  1. A *G*-test was used to evaluate the *Homogeneity* and *Topology* effects;
  2. A $\chi ^2$-test was used to evaluate the *Expertise* effect, while a *G*-test was used to evaluate the *Topology* effect (the *G*-test is recommended instead of the standard $\chi ^2$-test when more than $20\%$ of the cells have expected frequencies of less than 5).
- ***TTS and TIS.***
  1. Since the normality assumption was not met for $\mathrm {TTS}$, we analysed the *Homogeneity* effect through the Kruskal–Wallis statistical test instead of an ANOVA. Since in some topologies (e.g., the path and ring graph) $\mathrm {TTS}$ could not be computed in 4 out of the total 5 trials, the topology effect could not be evaluated. For $\mathrm {TIS}$, the normality assumption was met but the data were not homoscedastic. We ran the Welch’s ANOVA on $\mathrm {TIS}$, together with Games–Howell post-hoc tests for pairwise comparisons.
  2. Since the normality assumption was met neither for $\mathrm {TTS}$ nor $\mathrm {TIS}$, we used the Mann–Whitney test instead of a *t*-test.

**Parameter setting of the models (1)–(3).** See Supplementary Information.

## Supplementary information

Supplementary information

## Data Availability

Bardy, B. (2020, March 27). Moving in unison after perceptual interruption. Retrieved from osf.io/er8x5.
