# Supplementary material for: Moving in unison after perceptual interruption
Source: Sci Rep. 2020 Oct 22;10:18032. doi: 10.1038/s41598-020-74914-z (PMC7582161; doi:10.1038/s41598-020-74914-z)
Supplement: Supplementary file 1 — Supplementary information [file 41598_2020_74914_MOESM1_ESM.pdf]

Supplementary Information

Moving in unison after perceptual interruption

Benoît G. Bardy, Carmela Calabrese, Pietro De Lellis, Stella Bourgeaud, Clémentine Colomer, Simon Pla, Mario di Bernardo

Methods

A. Experimental conditions details

A..1 Topologies

Each group performed the experiments in four different interaction patterns among players (i.e., *topologies*), implemented through the combination of the spatial location of each participant and the use of home-made goggles limiting the field of vision to the desired location (see Fig. S1). Namely, the four topologies were

- **Complete graph**: participants had all the other players in their field of vision;
- **Ring graph**: each participant could only see the motion of the pendulums of their two closest neighbors;
- **Path graph**: this topology is similar to the ring graph, with the exception of two participants who could visualize the motion of only one neighbor;
- **Star graph**: this topology prescribes the presence of a hub, that is, a player who could see the motion of all the other players, who, in turn, could only see the motion of the hub.

B. Parameter setting of the models (1)-(3) of the main document

Selection of coupling strength c

For a given group and topology, we varied c between 0 and 1 with step 0.01 and, for each value of c, we ran 50 simulations of the Static Coupling (**SC**) model described in equation (1) of the main document. Each of the 50 simulations differed for the selection of the frequency ω_i and for the initial phase $\theta_i(0)$, $i = 1,\dots,7$. Specifically, the frequency of the i-th player was extracted from a Gaussian distribution with the mean and variance corresponding to the sample estimation performed in EC_1. The initial phases were selected from a uniform distribution in $[0,2\pi]$. For each value of c, we computed the average order parameter $\bar{r}(c)$ in the 50 corresponding trials. Then, we chose c as

$$\arg\min_{c} |\bar{r}_{\text{exp}} - \bar{r}(c)|, \tag{8}$$

where $\bar{r}_{\text{exp}}$ is the mean order parameter in EO across all the trials for the selected group and topology. The procedure was iterated to associate a value of c to each group and topology, see Table S1.

Selection of decay time τ

For each group, we varied τ such that $1/\tau$ ranges between 0.02 and 1 with step 0.02 and, for each value of τ, we ran 50 simulations of the **IM** model (equation (2) in the main document) differing for the initial phases and natural frequency of the player (selected as above). For each value of τ, we computed the average time in synchronization $\overline{\text{TIS}}(\tau)$ in the 50 corresponding trials. Then, we chose τ as

$$\arg\min_{\tau} \left|\overline{\text{TIS}}_{\text{exp}} - \overline{\text{TIS}}(\tau)\right|, \tag{9}$$

where $\overline{\text{TIS}}_{\text{exp}}$ is the mean TIS across all trials where TIS was statistically different from the TIS obtained from simulations of the **SC** model (Mann-Whitney test). The procedure was iterated to associate a value of τ to each group. The same steps were followed to tune τ in the Social Memory **SM** model (equation (3) of the main document). All the identified values of τ in the **SC** and **SM** models are reported in Table S4.

C. Comparing IM and SM models

For both models, and for all of the four topologies top $\in$ {Complete, Path, Ring, Star}, we computed the error $e_{\text{top}} = \left|\overline{\text{TIS}}_{\text{exp,top}} - \overline{\text{TIS}}_{\text{sim,top}}\right|$ where $\overline{\text{TIS}}_{\text{exp,top}}$ is the average TIS observed in the experiments, while $\overline{\text{TIS}}_{\text{sim,top}}$ is the average TIS obtained from the simulations. To evaluate the model that better fitted the data, a $t-$test was then run to assess the differences between the values of e_{top} observed in the **IM** and **SM** models, which are reported in Table S5.

Table S1. Coupling gains in Experiments 1 and 2.

	c Complete	c Path	c Ring	c Star
Experiment 1				
Matched	0.04	0.20	0.16	0.28
Matched-but-one	0.07	0.20	0.11	0.38
Natural	0.08	0.41	0.10	0.72
Experiment 2				
Dancers 1	0.08	0.43	0.10	0.50
Dancers 2	0.12	0.40	0.09	0.50
Non dancers 1	0.07	0.14	0.07	0.25

Table S2. Time-To-Synchronization (TTS) after eyes opening and Time-In-Synchronization (TIS) after eyes closing in Experiments 1 and 2.

	Mean TTS	Mean TIS
Experiment 1		
Matched	8.66 s	9.95 s
Matched-but-one	8.18 s	8.20 s
Natural	6.25 s	5.32 s
Experiment 2		
Dancers	8.99 s	8.81 s
Non dancers	7.21 s	6.26 s

Table S3. Experimental and Simulated Time-In-Synchronization (TIS); $^{**}p < 0.01$, $^{***}p < 0.001$.

	Mean TIS Exp	Mean TIS Sim
Experiment 1		
Matched	9.95 s	6.52 s**
Matched-but-one	8.20 s	5.94 s**
Natural	5.32 s	4.74 s
Experiment 2		
Dancers	8.81 s	5.92 s***
Non dancers	6.26 s	5.66 s

Table S4. Decay time τ estimated from data for each group and memory model in Experiments 1 and 2.

	τ_{IM}	τ_{SM}
Experiment 1		
Matched	12.50	8.33
Matched-but-one	10	12.50
Experiment 2		
Dancers 1	8.33	6.25
Dancers 2	8.33	8.33

Table S5. Experimental and Simulated Time-In-Synchronization (TIS) for each group, memory model and topology (in Path Matched-but-one, players did not stay in sync for at least 3 consecutive periods of length $2\pi/\omega_{\text{group}}$).

	$\overline{\text{TIS}}_{\text{exp}}$	$\overline{\text{TIS}}_{\text{IM}}$	$\overline{\text{TIS}}_{\text{SM}}$
Experiment 1			
Complete Matched	9.41 s	9.64 s	9.31 s
Complete Matched-but-one	9.02 s	9.27 s	8.31 s
Path Matched	12.50 s	8.60 s	8.31 s
Path Matched-but-one	No-sync	No-sync	No-sync
Ring Matched	4.90 s	9.14 s	8.88 s
Ring Matched-but-one	7.39 s	7.22 s	6.23 s
Star Matched	10.39 s	10.86 s	10.98 s
Star Matched-but-one	7.78 s	8.16 s	9.91 s
Experiment 2			
Complete D1	8.98 s	8.60 s	6.98 s
Complete D2	13.79 s	10.58 s	9.28 s
Path D1	7.53 s	7.83 s	6.95 s
Path D2	6.56 s	7.99 s	9.82 s
Ring D1	6.23 s	6.43 s	5.92 s
Ring D2	6.38 s	5.75 s	6.76 s
Star D1	7.88 s	7.40 s	8.65 s
Star D2	9.56 s	10.58 s	11.68 s

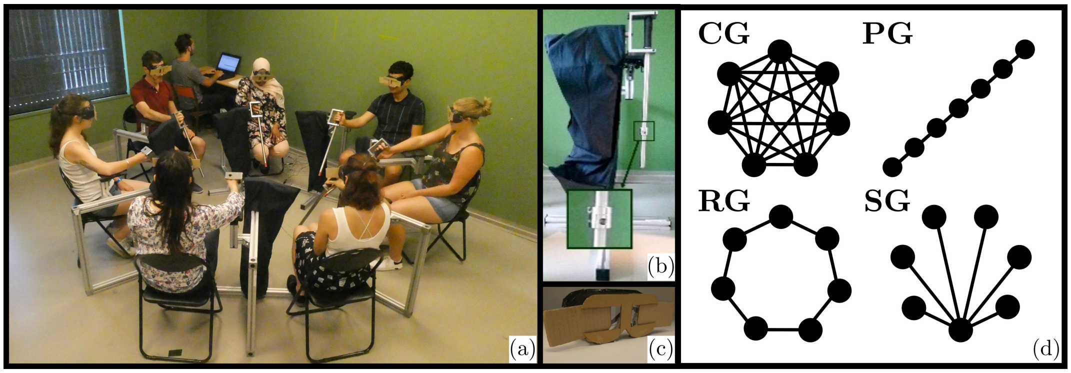

Figure S1. Experimental set up with (a) seven participants, (b) details on one aluminium pendulum showing the additional mass, (c) in-house goggles controlling the field of view; (d) complete, ring, path, and star graphs tested.

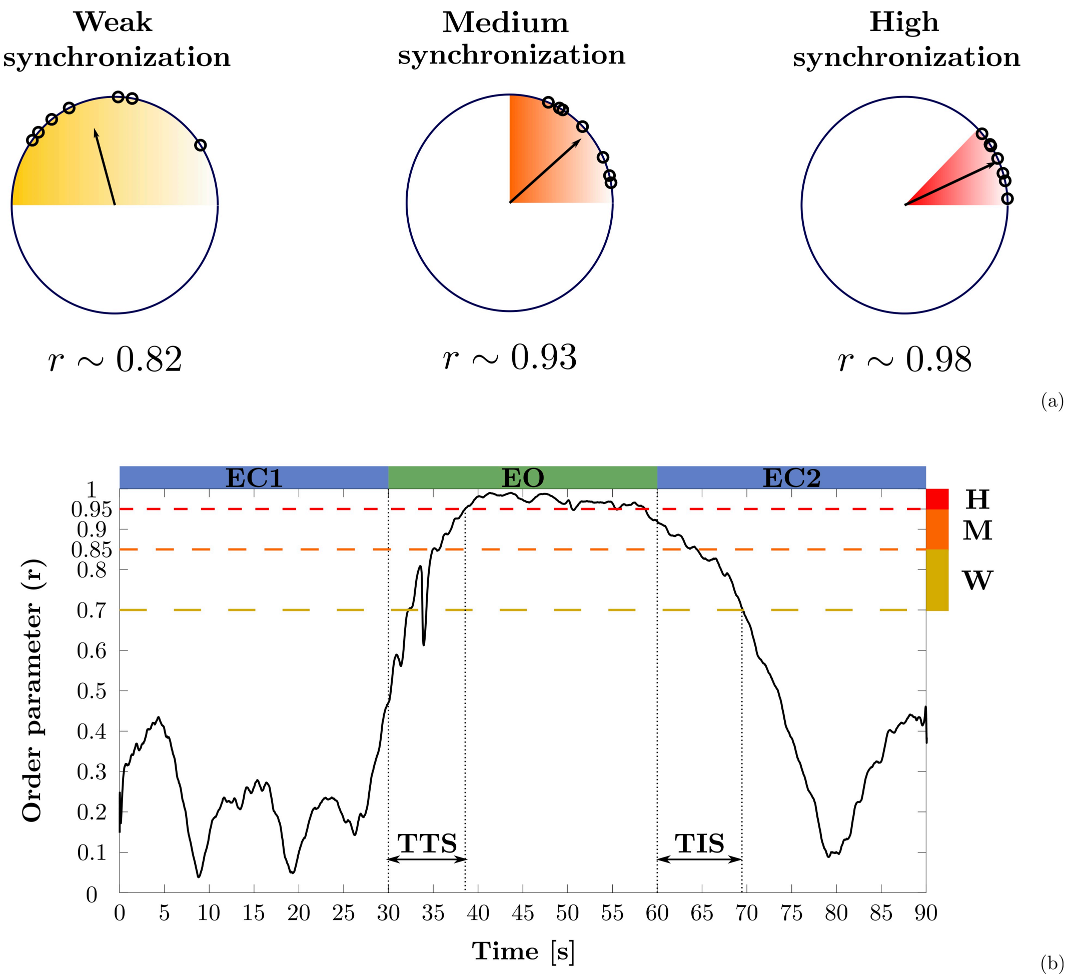

Figure S2. Three levels of synchronization — Weak (W), Medium (M), and High (H) — characterized by the value of the order parameter r, used to determine Time-To-Synchronization (TTS) and Time-in-Synchronization (TIS). *EO*: Eyes Open; *EC*: Eyes Closed.
